# A Comprehensive Review on Porcine Reproductive and Respiratory Syndrome Virus with Emphasis on Immunity

**DOI:** 10.3390/vaccines12080942

**Published:** 2024-08-22

**Authors:** Jorian Fiers, Ann Brigitte Cay, Dominiek Maes, Marylène Tignon

**Affiliations:** 1 Unit Viral Re-Emerging, Enzootic and Bee Diseases, Department Infectious Diseases in Animals, Sciensano, Groeselenbergstraat 99, 1180 Ukkel, Belgium; 2 Unit of Porcine Health Management, Department of Reproduction, Obstetrics and Herd Health, Faculty of Veterinary Medicine, Ghent University, Salisburylaan 133, 9820 Merelbeke, Belgium; dominiek.maes@ugent.be

**Keywords:** PRRSV, immunology, innate immunity, adaptative immunity

## Abstract

Porcine reproductive and respiratory syndrome virus (PRRSV) is one of the most important pathogens in pig production worldwide and responsible for enormous production and economic losses. PRRSV infection in gestating gilts and sows induces important reproductive failure. Additionally, respiratory distress is observed in infected piglets and fattening pigs, resulting in growth retardation and increased mortality. Importantly, PRRSV infection interferes with immunity in the respiratory tract, making PRRSV-infected pigs more susceptible to opportunistic secondary pathogens. Despite the availability of commercial PRRSV vaccines for more than three decades, control of the disease remains a frustrating and challenging task. This paper provides a comprehensive overview of PRRSV, covering its history, economic and scientific importance, and description of the viral structure and genetic diversity. It explores the virus’s pathogenesis, including cell tropism, viral entry, replication, stages of infection and epidemiology. It reviews the porcine innate and adaptative immune responses to comprehend the modulation mechanisms employed by PRRS for immune evasion.

## 1. The Porcine Reproductive and Respiratory Syndrome—History and Relevance

### 1.1. History

In the late 1980s, severe disease outbreaks caused by an unknown agent were reported in the United States’ swine production system. These outbreaks were characterized by reproductive losses in late gestational sows (mummified, stillborn and aborted fetuses) and the development of respiratory disease in young piglets, resulting in pneumonia, growth reduction and increased mortality [1,2]. Disease outbreaks with similar characteristics were reported in 1990 in Europe, but at that time, no link was found with the US outbreaks [3]. The disease was initially described by several names, including the mystery swine disease, the swine infertility and respiratory syndrome and the blue-ear pig disease. Eventually, a consensus name for the disease was found, based on the clinical signs that were observed in the field: the porcine reproductive and respiratory syndrome (PRRS) [4].

In 1991, the causative agent of the European disease outbreaks was isolated by researchers Terpstra and Wensvoort in porcine alveolar macrophages (PAMs) [5]. The isolated pathogen was an unrecognized RNA virus, and the isolated strain was named after their veterinary institute: the Lelystad strain [5,6]. One year later, North American researchers Collins et al. successfully isolated the PRRS-virus (PRRSV) from diseased pigs originating from a Minnesota swine herd, by cultivating tissue homogenates on the continuous cell line CL-2621 [7]. The isolated viral strain was designated as the VR-2332 strain. A comparison of the structural protein-coding sequences of these two reference strains revealed that both strains shared a certain homology in their open reading frames (ORFs), both with each other and with other viruses belonging to the *Arteriviridae* family. However, only a low degree of nucleotide identity was observed in the ORFs between the Lelystad strain and the VR-2332 strain, ranging from 55% (ORF5) to 79% (ORF6) [8]. Additionally, in a study by Allende et al., distinct differences were observed in the non-structural protein coding regions of the Lelystad strain in comparison to a North American isolate (16244B) [9]. These findings already suggested that the European and North American PRRSV strains belong to distinct genotypes, which will be further discussed in the next section. Interestingly, retrospective serological studies showed that antibodies against PRRSV were already present in sera collected from Canadian herds as early as 1979 [10], North American and South Korean herds in 1985 [11,12] and East-German herds in 1987 [13]. The results of these retrospective studies suggest that PRRSV was already circulating in herds worldwide for some years prior to the first reported clinical outbreaks. Ultimately, the true origin of the virus remains unclear. However, given the clear genetic differences between the European and North American strains, it is likely that PRRSV had a divergent evolution on the two continents, with both PRRSV strains originating from a distant common ancestor [14]. According to Plagemann (2003), the two distinct PRRSV strains might have originated from a closely related arterivirus, which was present in mice: the lactate dehydrogenase-elevating virus (LDV) [15]. In his hypothesis, a mutant LDV could infect European wild boars, which acted as an intermediate host. Following the importation of wild boars to North Carolina in 1912, this mutant LDV entered North America. Finally, a simultaneous but distinct evolution of the virus occurred for decades in both the European and North American wild boar populations until the virus was introduced in domestic pigs. Alternatively, genetic analyses performed by Stadejek et al. (2006) suggest that PRRSV was already circulating in Eastern Europe prior to the first reported Western outbreak [16]. Due to the political changes in the late Eighties, notably the fall of the Berlin Wall in 1989, the spread of the virus to Western Europe was facilitated. Finally, Hanada et al. (2005) speculated that PRRSV was transmitted from another, unknown, host species to the domestic swine population in about 1980 [17]. Afterward, the viruses explosively increased among domesticated swine due to an unprecedented high mutation rate, with a positive selection of alterations in the transmembrane regions of ORF5 that affected virus adaptation to the swine cell membrane [17].

### 1.2. Economic and Scientific Relevance

Three decades after the first reported outbreaks, PRRSV is endemic in most of the pig-producing countries, except Australia, New Zealand, five European countries (Norway, Sweden, Finland, Switzerland and Hungary since 2022), three South American countries (Argentina, Brazil and Chile) and Cuba, who are considered to be free of PRRSV [18,19]. Several studies have attempted to assess the economic relevance of PRRSV by modeling the potential losses caused by the virus. Holtkamp et al. (2013) estimated that PRRSV is responsible for an annual cost of 664 million US dollars, due to productivity losses, in all national breeding and growing-pig herds in the USA [20]. This is an 18.57% increase from the 560 million US dollar cost, due to PRRSV production losses, estimated by Neumann et al. in 2005 [21]. In a study by Nathues et al. (2017), an economic model, capable of estimating the costs of PRRSV on individual farms, was designed [22]. This model can be personalized for key herd factors including the used production system, type of batch farrowing, length of suckling period, production performance, disease status and more. The authors modelled the median annual loss per sow in a farrow-to-finish herd of 1000 sows for different severities of PRRS disease scenarios. The model estimated a median annual loss of €127 per sow if PRRSV would only slightly affect the reproductive system, while a median annual loss of €650 per sow is estimated when PRRSV severely affects both respiratory and reproductive systems. A similar estimation was made for fattening pigs: the estimated loss ranges from €3.77 per fattening pig with slight respiratory problems due to PRRSV to €17 per fattening pig with severe respiratory problems due to PRRSV.

In addition to the economic relevance of the disease, the scientific interest in PRRSV is obvious when looking at the number of scientific articles published since its discovery. A study comparing the publication counts in 1966 until 2016 for all common swine pathogens, reviewing more than 57,000 publications, ranked PRRS in sixth place in terms of having the most publication counts [23]. Additionally, PRRSV was the fourth most researched swine virus, ranked just below Influenza virus, Aujeszky’s disease virus (ADV) and foot and mouth disease virus, with the latter two pathogens being present in the swine herds for decades before the first reported PRRSV outbreaks.

## 2. Taxonomy, Viral Structure and Genetic Diversity

### 2.1. Taxonomy

PRRSV is classified in the order *Nidovirales*, family *Arteriviridae*, subfamily *Variarterivirinae* and genus *Betaarterivirus* [24]. Other families belonging to the order *Nidovirales* include the *Tobaniviridae*, *Roniviridae* and *Coronaviridae*. The latter family gained a lot of attention in recent years due to one of its members, the severe acute respiratory-syndrome-related SARS-CoV-2, responsible for the COVID-19 pandemic [25]. The *Arteriviridae* family consists of six subfamilies, 13 genera, 11 subgenera, and 23 species, of which only a few members are well characterized. These members include the equine arteritis virus (EAV), the simian hemorrhagic fever virus, the wobbly possum disease virus, LDV and PRRSV [26,27]. Given the clear genetic differences between the European and North American strains, as described in the previous section, PRRSV has been classified into two distinct species: Betaarterivirus suid-1 and Betaarterivirus-suid 2 [24]. The former is better known as PRRSV-1 and is mainly present in Europe, with the Lelystad strain considered as the reference strain. The latter is known as PRRSV-2 and is the predominant species in North America and Asia, with the VR-2332 as the reference strain [24]. Throughout this introduction, both species will be collectively referred to as PRRSV. If a distinction between both species is needed, they will be referred to as PRRSV-1 and PRRSV-2.

### 2.2. Genome and Viral Structure

**Figure 1:**
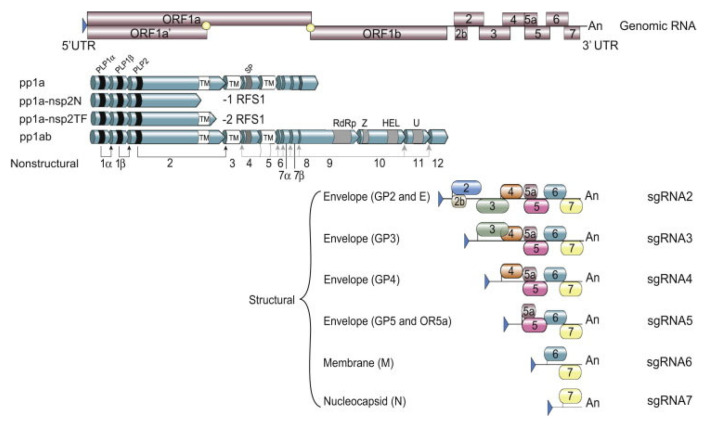
Schematic overview of the PRRSV genome with the transcription and translation mechanism (from [29]). UTR = untranslated region, ORF = open reading frame, pp = polyprotein, RFS = ribosomal frame shifting, nsp = non-structural protein, GP = glycoprotein, sgRNA = subgenomic RNA.

Like all other members belonging to the order *Nidovirales*, PRRSV is an enveloped, positive-stranded RNA virus. Members belonging to the *Arteriviridae* family have the smallest genome size in the order *Nidovirales*, with genome sizes ranging from 12.7 to 15.7 kb in length [28]. The PRRSV genome size ranges from 14.9 kb to 15.5 kb and consists of 10 ORFs with a 5’ Cap untranslated region (UTR) and a polyadenylated 3’ UTR (Figure 1) [29].

**Figure 2:**
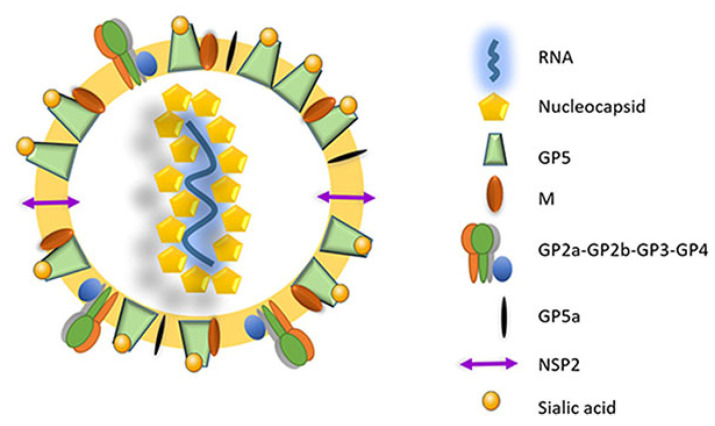
The mature PRRSV virion consists of an internal nucleocapsid complex (nucleocapsid proteins + viral RNA) surrounded by a lipid bilayer envelope containing the different structural proteins (adapted from [42]). GPx: glycoprotein x; M: membrane protein; NSP2: non-structural protein 2.

The virus utilizes two different transcription mechanisms for the expression of both non-structural and structural proteins: ribosomal frameshifting (RFS) and the synthesis of subgenomic RNA (sgRNA), respectively. The non-structural proteins are all encoded from ORF1a and ORF1b. In short, these two ORFs, both located at the 5′ end of the genome, translate into four different non-structural polyproteins (pp). These include pp1a, pp1a-nsp2N, pp1a-nsp2TF and pp1ab and are generated by the mechanism of RFS [30,31,32]. The simplified principle of the RFS mechanism, which was first described by Jacks and Varmus in 1985 [33], is the induced movement of ribosomes to the −1 reading frame (in the direction of the 5′ end) at which the ribosomes continue the translation in this new reading frame [34]. Eventually, the four generated polyproteins are autocatalytically processed into 16 different non-structural proteins (NSP) via a combination of co- and post-translational modifications, with the help of RFS and four virally encoded proteinases (papain-like cysteine proteinases—PLP1α, PLP1β, PLP2 and the serine proteinase—SP) [35]. Most of the generated NSPs assemble into a large replication and transcription complex (RTC), with, as the most important components, the RNA-dependent RNA polymerase (RdRp—NSP9), the zinc-binding domain (NSP10), the RNA helicase (NSP10) and the conserved nidovirus uridylate-specific endoribonuclease (NSP11). The assembled RTC is in turn responsible for both the replication of the viral genome and the synthesis of a set of six sgRNAs, which are produced by the generation of a negative-strand intermediate [36,37,38]. These six sgRNAs express eight different ORFs, which are in turn responsible for encoding the eight different PRRSV structural proteins. In this way, glycoprotein (GP) 2 and the unglycosylated, envelope protein (E) are encoded by ORF2a/b, which is expressed by sgRNA2. GP3 and GP4 are encoded by ORF3 and ORF4, respectively, which are expressed by sgRNA3 and sgRNA4. The sgRNA5 expresses two ORFs, ORF5 and ORF5a, which translate into GP5 and GP5a, respectively. ORF6 is expressed by sgRNA6, and the translation of ORF6 results in the expression of the membrane protein (M), which is the most highly conserved structural protein of PRRSV [39]. Finally, ORF7 is expressed by sgRNA7 and is responsible for the translation of the nucleocapsid protein (N) (Figure 1) [29]. Half of the viral proteins are GP5 and M proteins and due to this, these two proteins are considered the major envelope proteins. The remaining structural proteins, except the N protein, are considered minor envelope proteins [40]. At the end of the replication cycle, the generated structural proteins assemble into the mature PRRSV virion (Figure 2). First, the nucleocapsid complex is formed via interaction between the synthesized N proteins and the synthesized genomic RNA molecule. Second, the nucleocapsid complex acquires the viral envelope, consisting of the remaining seven structural proteins. Interestingly, Kappes et al. (2013) have shown that multiple isoforms of the NSP2 can also be incorporated into the mature virion [41]. The complete PRRSV virions are spherical to oval-shaped, have a smooth surface and a median diameter of approximately 55 nm when visualized using negative stain electron microscopy [40].

### 2.3. Genetic Diversity and Phylogeny

PRRSV has a high degree of genetic diversity, which arises from random point mutations, genome rearrangements and selection [43,44]. Mutations are the result of random errors that occur by the PRRSV RdRp during replication. Because the RdRp of the members of the *Arteriviridae* family lacks the capability of a 3′ proofreading, these random errors are not corrected, and consequently, the rate of introduction of these point mutations is very high [45]. For PRRSV, it is estimated that at least one point mutation is introduced during each replication cycle [46]. Additionally, recombination events are widely documented for PRRSV, resulting in the production of mosaic isolates [47,48]. In the case of recombination, parts of the genome of different PRRSV strains can be exchanged when more than one PRRSV strain simultaneously infects the same pig. The mechanism behind the recombination events has been explained by the copy-choice or template switching model. During the transcription of sgRNAs, the RdRp can switch from one RNA template to another (template switching), especially in PRRSV genomes that already have a high degree of similarity [47,49].

The immense genetic diversity of PRRSV is reflected in the complexity of phylogenetic analyses. Historically, the PRRSV-1 strains have been classified into four different subtypes with the West-European subtype 1 being further classified into twelve distinct clades [16,50,51]. The PRRSV-2 strains have been classified into nine lineages, which include five large clusters and four smaller groups of strains [52]. These phylogenetic analyses were mainly based on sequencing of the ORF5, given the high genetic variability of this genomic region [50]. However, the ORF5 sequence accounts for only 4% of the total PRRSV genome, and important genetic information residing in the other regions of the PRRSV genome is missed when only targeting this region for phylogenetic analysis. This is especially important for the detection of recombinant strains, since PRRSV does not contain typical hot-spots in which recombination events occur preferably [48]. Given the recent advancements in sequencing technology, which allow the rapid whole-genome sequencing of PRRSV, the ORF5 sequencing will increasingly be replaced by an analysis of the full PRRSV genome [48,53,54].

The observed genetic diversity is also reflected in the clinical manifestation of the disease. During the 30 years since the first reported outbreaks, several highly pathogenic PRRSV (HP-PRRSV) strains have emerged worldwide, which has led to acute disease outbreaks with high mortality. In the second half of the 1990s, an atypical and acute PRRSV-2 variant appeared in the United States, causing abortion storms and high mortality [55,56]. An HP-PRRSV type 2 strain, which emerged around 2006 in Asia, was responsible for porcine high fever disease, causing severe respiratory pathology and associated high mortality in young and old pigs [57]. In Eastern Europe, a Belarusian highly pathogenic subtype 3 PRRSV-1 isolate (Lena strain) was responsible for severe production losses, with both a high incidence of abortions and mortality rates up to 70% in the growing pigs [58]. In Italy, an HP-PRRSV-1 subtype 1 (PR40 strain), capable of strongly modulating the immune system, was isolated from a commercial herd suffering from an atypical PRRS outbreak [59,60]. Recently, outbreaks of PRRSV-1 strains with increased virulence have been reported in Spain, causing abortion rates up to 27%, fertile sow mortality up to 6.5% and up to 50% mortality in nurseries [61]. Finally recombinant PRRSV strains, both PRRSV-1 and PRRSV-2 strains, with increased virulence and clinical signs have been reported in China, France and Denmark [62,63,64,65].

## 3. Pathogenesis

### 3.1. Cell Tropism, Viral Entry and Replication

PRRSV has a very narrow in vivo cell tropism, a shared characteristic with other members of the *Arteriviridae* family (except of EAV) [35]. Moreover, domestic swine, feral swine, and wild boars are the only known species that support a natural PRRSV infection. Susceptible cells for in vivo infection include cells of the monocyte and macrophage lineages, with infection primarily occurring in subsets of macrophages present in the lung, placenta and lymphoid tissues [66]. Some Lineage 1 PRRSV-2 strains have recently emerged with gained tropism in the small intestine [67,68]. The main target cells for in vivo viral replication are the porcine alveolar macrophages (PAMs) [69]. The in vitro study of PRRSV should ideally be performed in primary cultures of PAMs to mimic the in vivo kinetics of infection as much as possible [66]. However, continuous cell lines, including the African green monkey kidney cell line MA-104 and derivatives of this cell line (MARC-145 and CL2621), are permissive to PRRSV infection as well [35,70]. Alternatively, the generation of a continuous PAM cell line provides a valuable tool for the in vitro study of PRRSV [71].

The narrow in vivo tropism of PRRSV is caused by the specific interactions between the PRRSV structural proteins and the cellular receptors on the host cells. The scavenger receptor CD163, mainly expressed on macrophages and monocytes, has been identified as the vital factor for PRRSV infection given its ability to promote viral uncoating and internalization [72,73]. Several studies have shown that the presence of CD163 is a necessity for PRRSV infection. Firstly, two independent studies with gene-edited pigs showed that pigs lacking the scavenger receptor cysteine-rich domain 5 (SRCR5) of the CD163 receptor are protected from PRRSV infection [74,75]. More recently, the gene edition has been restricted to a point mutation in CD163 (E529G) with similar results [76]. Secondly, Xu et al. (2020) have shown that monoclonal antibodies against the SRCR5-9 can block different PRRSV stains in a dose-dependent manner [77]. In addition to CD163, the interaction between sialoadhesin (Sn/CD169/Siglec-1) and the PRRSV M/GP5 complex has been described to mediate the internalization of the virus in the host cell [78,79]. An in vitro infection study in non-permissive PK-15 cells, conducted by Van Gorp et al. (2008), showed that the co-expression of CD163 and Sn produced ten to hundred times more viral particles compared to the expression of CD163 alone [72]. However, in contrast to CD163, Sn is not required for in vivo PRRSV infection, since Sn-edited pigs remain PRRSV susceptible [80]. Additionally, heparin sulfate has been identified as an attachment factor for PRRSV by interacting with the M protein [81,82]. Several other host receptors and proteins have been described to interact with PRRSV, including CD151, vimentin, DC-SIGN, MYH9, Siglec-10, heat-shock proteins or macrophage receptor with collagenous structure (MARCO). Furthermore, Rowland and Brandariz-Nuñez (2024) highlighted the role of N-glycans from PRRSV in the viral infection and, more precisely, in regulating viral entry [83]. More research is warranted to fully understand their role during PRRSV infection [84,85,86,87]. Finally, it has been shown that PRRSV can use viral apoptotic mimicry as an alternative pathway for infection by externalizing phosphatidylserine on its viral envelope. Consequently, the T-cell immunoglobulin and mucin domain 4 (TIM-4) of PAMs will recognize the PRRSV virion as apoptotic debris, leading to the induction of micropinocytosis and the uptake of the PRRSV virion [85].

After viral attachment, PRRSV enters the permissive cell through the process of clathrin-mediated endocytosis, a shared characteristic with other members of the *Arteriviridae* family [35,88]. Once the virus has been internalized, the uncoating of the viral particle occurs via a combination of endosome acidification and membrane fusion, which is followed by the translation of the released viral genome into the cytoplasm, as described in Section 2.2 [89]. By the end of the replication cycle, the newly synthesized N proteins package the replicated full-length RNA genomes into the nucleocapsid complex, and this complex acquires the viral envelope proteins via budding of the Golgi complex. The newly generated viral particles accumulate in the cytoplasm of the host cell and are eventually released from the infected cell by exocytosis [38].

### 3.2. Stages of Infection, Disease Mechanisms and Clinical Signs

#### 3.2.1. Acute and Persistent Infections

In general, three distinct stages can be observed during PRRSV infection: the acute, persistence, and extinction stage [89]. The acute stage of infection occurs during the first weeks following exposure to the virus. The initial viral replication takes place in the susceptible tissue-resident macrophages of the nasal mucosa, which is followed by a rapid spread of the virus using the lymph-hematic route [90]. PRRSV can be detected in blood and lung tissue by 6–48 h after exposure, and viral loads reach their peak at 4–14 days post-infection (dpi), depending on the infecting strain and the pre-existing immunity [18]. Peak viral titers in the serum of infected animals usually rise to 10^5^ tissue culture infectious dose 50%/mL (TCID 50/mL), but in the case of HP-PRRSV strains, viral titers of more than 10^8^ TCID 50/mL have been reported [91,92]. Interestingly, there is a certain correlation between the age of the animals at exposure and the observed viral titers: the younger the pigs are at exposure, the higher the viral titers are and the longer the viremia lasts [93,94]. Most of the observed clinical signs occur during the acute phase when viral titers reach their peak. Once the viral peak has been reached, a rapid decrease of the viral load is usually observed, with most pigs being no longer viremic by 21–28 dpi [18]. The persistence of infection is a characteristic that has been described for other members of the *Arteriviridae* family, including LDV and EAV [95,96]. In PRRSV-infected pigs, the virus can remain present in the body for months after the last clinical signs are observed. In a study by Wills et al. (1997), PRRSV was isolated from oropharyngeal samples of persistently infected pigs up to 157 dpi [97]. Additionally, in a study by Bierk et al. (2001), persistently infected sows were able to transmit PRRSV to contact controls at 42 to 56 dpi [98]. Finally, when viral shedding no longer occurs, the extinction stage begins [18]. The duration of this stage is dependent on both the genetic background of the pig and the PRRSV strain. The replication of PRRSV has been described for as long as 250 dpi, meaning that in the case of a conventional production system, PRRSV infection can remain present during the whole period of fattening [89,99].

#### 3.2.2. Induction of Apoptosis

The main disease mechanisms during PRRSV infection include the induction of cell death, the modulation of the inflammatory response and the increased susceptibility to secondary infections. The induction and modulation of apoptosis by PRRSV, both in vitro and in vivo, has been described by several research groups [100,101,102,103]. Apoptosis is typically considered as the programmed process of cell death in which the cell dismantles in a structured manner without the induction of inflammation. This is in contrast to necrosis, which refers to the accidental, non-programmed cell death, which is influenced by environmental factors and leads to the release of inflammatory cellular contents [104]. The molecular mechanisms responsible for triggering apoptosis are complex and are divided into three main pathways: the extrinsic (death receptor), intrinsic (mitochondrial), and perforin-granzyme pathway. Nevertheless, these three pathways converge into the same execution pathway, characterized by the fragmentation of the cellular DNA, degradation of the cytoskeleton and cellular proteins, the formation of apoptotic bodies, and the final uptake by phagocytic cells [105]. During PRRSV infection, a modulation of both the extrinsic and intrinsic pathways has been described. This includes the upregulation of pro-apoptotic proteins and the downregulation of anti-apoptotic proteins, both involved in the mitochondrial pathway, and an increased expression of receptors involved in the death receptor pathway [106,107]. Interestingly, despite the increase in cell death, the virus is able to efficiently spread to neighboring cells. A possible mechanism to achieve this is the ability of PRRSV to transmit its viral RNA and structural proteins to neighboring cells using intercellular tunneling nanotubes (TNTs) [108,109]. The latter might be an additional contribution to the observed persistence of PRRSV in lung and lymphoid tissues [110]. Finally, next to the direct induction of apoptosis in infected cells, it has been shown that cells not (yet) infected with PRRSV can also become apoptotic, by a mechanism called bystander apoptosis [111,112].

#### 3.2.3. Modulation of Immune Response and Secondary Infections

Next to the induction of apoptosis, PRRSV can modify the immune response, which will be discussed in detail in the next sections. In the context of disease mechanisms, it is noteworthy to mention that increased secretion of proinflammatory cytokines, including tumor necrosis factor-α (TNF-α), Interleukin (IL)-1 and IL-6, has been reported in pigs infected with HP-PRRSV [113,114]. These proinflammatory cytokines increase the microvascular permeability, inducing the influx of leukocytes to the site of infection. This can eventually lead to pulmonary edema, resulting in respiratory problems [114]. Furthermore, the induced systemic effects of the inflammatory cytokines lead to an overall diseased stage, characterized by pyrexia, anorexia and lethargy. Both the depletion of pulmonary immune cells, by the increased incidence of cell death, and the modulated respiratory immune response make PRRSV-infected pigs more susceptible to secondary, opportunistic pathogens [89]. Since these opportunistic pathogens are often bacteria, PRRSV can indirectly lead to increased antimicrobial usage. Due to the increased susceptibility of PRRSV-infected animals to other pathogens, PRRSV is one of the most important contributors, together with *Mycoplasma hyopneumoniae*, to the porcine respiratory disease complex (PRDC) [115]. PRDC is a multifactorial respiratory syndrome in which pigs are infected with both primary and secondary respiratory pathogens and in which both infectious and non-infectious factors play a role in the eventual outcome [116]. Finally, several studies have shown the synergistic effect of PRRSV with other pathogens where a co-infection between PRRSV and the other pathogen exacerbates the observed clinical signs of the pathogens alone [117]. This has been shown for pathogens such as *Mycoplasma hyo-pneumoniae* [118], *Bordetella bronchiseptica* [119] and Porcine Circovirus Type 2 [120].

#### 3.2.4. Clinical Signs

The clinical signs and severity of clinical disease, observed during a PRRSV infection, depend on the production stage in which the pig is infected, the PRRSV strain which causes the infection, the pre-existing immunity of the pig and co-infections. In some cases, PRRSV can remain subclinical without evident disease signs. In other cases, especially during infection with HP-PRRSV, high morbidity and mortality can be observed. Taken together, infected piglets and fattening pigs mainly show clinical signs of respiratory distress. These signs include coughing, sneezing and dyspnea and can lead to growth reduction and increased mortality [18]. Still, occasional diarrhea due to severe hemorrhagic injuries of the small intestine has been reported in piglets [121,122]. In infected gilts and sows, these respiratory problems can also be observed. However, the main clinical manifestations of PRRSV in breeding animals are related to reproductive failure and are dependent on the gestation stage at which infection occurs [5,56,123,124,125,126,127,128,129] as reviewed by [130]. No interference with fertility has been observed at the onset of gestation in case of infection [126]. During early gestation, a low and non-pathologic incidence of embryonic infection has been described after PRRSV inoculation of gilts at 14 days of gestation [125]. During mid-gestation, PRRSV inoculation of gilts and sows did not lead to PRRSV infection of the fetuses, and consequently, no pathology was observed [124,127]. Finally, the main clinical manifestation of the disease is observed when gilts or sows are PRRSV-infected during late gestation [5,56,127,128,129]. At that point, PRRSV can easily replicate and cause pathology in both the endometrium and placenta, resulting in the transplacental infection of the fetuses and the consequent signs of reproductive disease [123,127,130]. The main reason for the correlation between the gestation stage and susceptibility to transplacental infection likely resides in the presence of PRRSV-susceptible cell populations in the endometrium and placenta. In short, CD163+Sn+ cells are present in the endometrium during all gestation stages. However, Sn+ cell populations have only been detected in placental tissue collected during early gestation (20–35 days) and late gestation (70–80 days and 114 days) and not in placental tissue collected during mid-gestation (50–60 days). Moreover, the number of Sn+ cells was lower at 20–35 days and 70–80 days compared to the number of Sn+ cells at 114 days, which further contributes to the higher susceptibility for transplacental infection during late gestation [131].

PRRSV-infected boars can also show signs of respiratory distress and an overall diseased status. However, the male reproductive function is also seriously altered with infection of the male reproductive organs. The virus replicates in the epithelial germ cells of the seminiferous tubules, primarily spermatids and spermatocytes, and in the macrophages, which are in the interstitium of the testis [132,133]. The infected Sertoli cells and spermatogonia undergo apoptosis during early differentiation, creating damage in the blood-testis barrier, and disrupting the androgen secretion with a significant reduction of testosterone and anti-Müllerian hormone levels [134]. After infection, the PRRSV is present in the semen of boars where it can persist for 25 to 92 days [135,136,137]. From economic and sanitary points of view, the most important consequences of PRRSV infection in boars include reduced semen quality and loss of libido as well as the potential transmission of PRRSV via the semen during artificial insemination (AI) of recipient sows [18,138].

### 3.3. Transmission, Risk Factors and Biosecurity

PRRSV infection of pigs can occur by both direct contact with other infected animals or by indirect contact with fomites containing infectious particles. Throughout PRRSV infection, the shedding of viral particles occurs mainly in the nasal secretions and saliva. However, shedding in urine, feces and mammary gland secretions has also been described. Importantly, viral shedding in semen is an important route of PRRSV introduction, especially in the context of AI. The main routes of introduction/exposure include aerial transmission, insemination with PRRSV-infected semen, ingestion and direct contact with fomites [139,140,141,142,143,144,145]. Several factors can influence PRRSV transmission, including the age of the animal, the infecting PRRSV strain and the immune response of the animal [90].

The main risk factors for the introduction of a PRRSV field isolate into a sow herd include the purchase of infected replacement gilts/sows and the purchase of semen from AI centers with infected boars [141,145]. To control these risk factors, adequate external biosecurity should be implemented. If possible, replacement animals should be purchased from PRRSV-free nucleus herds and semen from PRRSV-free AI centers. The purchased animals should be placed in a quarantine unit for at least 28 days before introduction into the herd. Ideally, this unit should be isolated from all animals and contain a separate entrance and separate hygiene lock [146]. During this period, the animals should be monitored for clinical signs of PRRS, and diagnostics should be performed to ensure that no PRRSV infection is present in the purchased animals. The main tools for diagnosing PRRSV include reverse transcription polymerase chain reaction assay (RT-PCR), for the detection of PRRSV RNA, and enzyme-linked immunosorbent assay (ELISA), for the detection of PRRSV-specific antibodies [147]. An additional risk factor for introduction is the airborne transmission of PRRSV from neighboring farms. Although the long-distance airborne transmission of PRRSV has been described [145,148,149,150], the probability of this phenomenon is relatively low [139,151,152,153]. Furthermore, an adequate air filter system can be used to mitigate this risk [154,155,156,157,158].

## 4. Modulation of the Innate Immune Response

### 4.1. Innate Immune Cells and Their Interaction with PRRSV

The innate immunity can be considered as the first line of defense against pathogens, and it is found in all multicellular organisms. Its role consists of the non-specific, fast (minutes to hours), recognition and attack of infectious pathogens. It can be divided into three large components, each with its specific functions and key players. The first component consists of physical barriers, such as the skin and mucous membranes, which are responsible for preventing the entrance of infectious agents. The second component includes chemical barriers, such as antimicrobial peptides, pH, certain lipids and specific enzymes. The main functions of these chemical barriers are the prevention of entry and chemical destruction of invading pathogens. Finally, the third component consists of the innate immune cells, which are further classified into distinct cell types and are responsible for pathogen recognition, clearance and the activation of the adaptive immune response [159]. In the context of viral infections, mainly macrophages, dendritic cells (DCs) and natural killer (NK) cells play a major role in the first line defense. After the detection of an invading pathogen, the tissue-resident macrophages and DCs are activated and start producing inflammatory cytokines and chemokines, resulting in the recruitment of additional immune cells, such as NK cells, neutrophils and monocytes [160].

#### 4.1.1. Macrophages

Macrophages are the main phagocytic cells of the innate immune system. They exhibit a broad variety of functions, including the engulfment and clearance of infecting pathogens, the removal of cell debris and harmful agents and the production of inflammatory cytokines and chemokines. The latter results in the recruitment of other immune cells to the site of infection, enhancing the immune response. A typical characteristic of macrophages is their heterogeneity and plasticity in terms of morphology, function and expression of surface antigens, which is dependent on their surrounding microenvironment. Macrophages can be classified into two main types, namely the yolk sac-derived tissue-resident macrophages and the monocyte-derived macrophages. The former have an embryonic origin and consist of a heterogeneous group with a functional specialization in different tissues. They reside within the tissues, in which they form a self-renewable population that is responsible for tissue surveillance and homeostasis. Tissue-resident macrophage populations can be found in the spleen, lymph nodes, intestines, tonsils, liver, lungs, nasal mucosa, skin, endometrium and placenta. The monocyte-derived macrophages originate from the bone marrow and are recruited and activated during infection. The monocytes, the macrophage progenitors, circulate in the blood and will migrate to the site of infection by reacting to inflammatory signals. Subsequently, they will differentiate into activated macrophages [161,162].

In the context of PRRSV infection, macrophages play a critical role as they are the main target for viral entry and replication. The first important macrophage subpopulations are in the nasal mucosa since this is the primary site of entry. The major subpopulation of nasal macrophages includes the CD163+Sn−, located in the epithelium and upper lamina propria, and the CD163+Sn+, located deeper in the lamina propria [163,164]. As stated in Section 3.1, CD163 is the necessary receptor for PRRSV attachment and viral entry, with Sn being the main mediator for efficient internalization of the virus. Interestingly, Oh et al. (2020) showed that the CD163+Sn- subpopulation had a 4.9 times higher susceptibility to the PRRV-1 subtype 3 strain Lena than the reference LV strain, which might be facilitated by the observed upregulation of CD163 in the Lena-inoculated cell cultures [164]. These results suggest that PRRSV is already able to infect the macrophages present in the upper epithelium, in a strain-dependent manner, before further infection of the CD163+Sn+ in the deeper layers of the lamina propria. As previously stated, the lungs contain the main target cell for PRRSV infection, the PAMs, which express high levels of both CD163 and Sn. A phenotypically similar macrophage subpopulation can be found in the lung parenchyma, the porcine intravascular macrophages (PIMs), which are also susceptible to PRRSV infection [165]. PIMs are localized in the blood vessel lumen and can directly shed virus in the blood circulation. Due to this, the PIMs might be the major cell type responsible for PRRSV viremia [166,167]. PRRSV-infected macrophages show a drastically reduced type I interferon response (see Section 4.2) and an aberrant cytokine production [168].

#### 4.1.2. Dendritic Cells

The DCs are a heterogeneous group of specialized cells, expressing a wide range of cell-surface markers. They are considered the major antigen-presenting cells (APCs), given their unique characteristic of being able to activate naive lymphocytes. Their main task is the uptake of antigens and the subsequent processing of these antigens into major histocompatibility complex (MHC)-peptide complexes. Following antigen uptake, they migrate towards the secondary lymphoid organs in which they present the MHC-peptide complex to both T-lymphocytes and B-lymphocytes, which induces their activation. In this way, the DCs can form a bridge between the innate and adaptive immune response. Moreover, they are involved in the activation and regulation of innate immune cells, including the NK cells and neutrophils. Additionally, they are further involved in the proliferation, differentiation and isotype switching of B-lymphocytes (see Section 5.2) via the production of B-cell stimulatory factors. DCs can be classified into two large subsets, based on functional and phenotypic differences.

- The first subset contains the conventional DCs (cDCs), which play a major role as APCs.
- The second subset consists of the plasmacytoid DCs (pDCs), which play a role in the regulation of the immune response, by production of both anti-viral cytokines (mainly IFN-α) and proinflammatory cytokines and chemokines, including IL-6, IL-12, CXC-chemokine ligand 8 (CXCL8), CXCL10, CC-chemokine ligand 3 (CCL3) and CCL4.

Despite this differentiation, both classes of DCs can interact with each other: cDCs can facilitate the maturation of the pDCs, while pDCs can increase the antigen presentation of cDC [169,170,171,172,173].

The interaction between PRRSV and DCs has been studied by several research groups [174,175,176,177,178,179,180,181] and reviewed by [182]. Early studies mainly focused on the interaction between PRRSV and monocyte-derived DCs (moDCs) or bone marrow-derived DCs (bmDCs) [175,181,183]. Both moDCs and bmDCs were able to support PRRSV infection and replication, resulting in an impairment of the antigen-presenting function, which suggested a role for DCs in the immunopathogenesis of PRRSV [174,175,180]. However, studies using either ex vivo harvested cDCs or tracheal explants were contradictory to the results gathered in the moDCs and bmDCs [176,177,178]. In both the studies by Loving et al. (2007) and Bordet et al. (2018b), PRRSV was not able to infect ex vivo lung cDCs [176,177]. However, the latter study did show a strain-dependent upregulation of IFN-α, IL-12, TNF-α and transforming growth factor-β (TGF-β) mRNA in lung cDCs exposed to a highly virulent PRRSV-1 strain (Lena, but not in lung cDCs exposed to a low virulent PRRSV-1 strain. Similar results were observed using tracheal explants: PRRSV was not able to infect the cDCs, but the exposure did induce the expression of cytokines (IFN-α and IL-10) [178]. In a recent study performed by Li and Mateu (2021), in vitro cDCs were not susceptible to infection by a moderate virulent PRRSV-1 strain and no sign of maturation nor cytokine expression was induced [179]. However, when these cDCs were exposed to infected cells (infected with the same PRRSV-1 strain), both the maturation of the cDCs and the expression of cytokines (IL-12 + IL-10) were observed. Finally, the pDCs are not susceptible to PRRSV infection. Nevertheless, modulation of the IFN-α response by PRRSV has been reported, which will be further discussed in Section 4.2 [184,185].

#### 4.1.3. Natural Killer Cells

NK cells are important components of the innate immune response against viral infections, by being responsible for the early cytotoxic killing of infected cells, which prevents further viral infection. Moreover, they can communicate with surrounding cells through the production of proinflammatory cytokines and chemokines, and they play an important role in the early production of IFN-γ. This cytokine increases the T-cell response, and, in this way, NK cells can enhance the adaptive immune response [160,186].

Several studies have described the reduction in NK cytolytic activity by PRRSV. In an in vitro study by Cao et al. (2013), a reduced cytotoxic killing of PRRSV-infected PAMs was observed in comparison to PAMs infected with the ADV [187]. Additionally, inactivated PRRSV was also capable of suppressing the NK function, albeit to a lesser degree than infectious PRRSV. In an in vivo study by Dwivedi et al. (2012), a 50% reduction in NK cell cytotoxicity was observed 2 days after PRRSV infection compared to the NK cell cytotoxicity before infection [188]. Finally, in vivo PRRSV challenge reduced the NK cell cytotoxicity by 50–80%, compared to the cytotoxicity observed in mock-challenged piglets. For comparison, a reduced cytotoxicity of only 10–30% was observed in pigs challenged with the porcine coronavirus (PRCV), compared to the same mock-challenged piglets. Moreover, dual infection of both PRRSV and PRCV had a synergistic effect, with a reduced NK cytotoxicity of 80–100%, in comparison to the mock-challenged pigs [189].

### 4.2. Pathogen Recognition and Type I Interferon Response

The innate immune cells need to be capable of distinguishing invading pathogens from host proteins. To do this, the innate immune cells contain a range of conserved pathogen recognition receptors (PRRs). These receptors can sense both different signature molecules expressed by pathogens, known as pathogen-associated molecular patterns (PAMPs), and molecules associated with cellular damage, known as damage-associated molecular patterns (DAMPs). Different classes of cellular PRRs are described, including the Toll-like receptors (TLRs), the nucleotide-binding oligomerization domain-like receptors (NLRs), the retinoic acid-inducible gene-I-like receptors (RLRs), the membrane C-type lectin receptors (CLRs) and the DNA receptors [190]. Within each class of PRRs, there a several receptors, and each receptor can sense a certain PAMP specific for a certain class of pathogens [191]. In the case of viral infections, the interaction between the viral PAMPs and the PRRs expressed by the host immune cells triggers a cascade of signaling pathways. These signaling pathways eventually lead to the antiviral response, consisting of the production of both proinflammatory cytokines and interferons [160].

PRRs capable of binding either viral ssRNA or viral dsRNA (intermediate during viral replication) can interact with PRRSV during infection. The main PRRs capable of sensing RNA viruses include three members of the TLRs, TLR3, TLR7, and TLR8, and two members of the RLRs, retinoic acid-inducible gene I (RIG-I) and the melanoma differentiation-associated antigen 5 (MDA5) [192]. In pigs, a high expression of the TLRs capable of sensing viral RNA is observed in both DCs and macrophages: TLR3 is mainly expressed in pDCs, TLR7 is mainly expressed in macrophages, pDCs and, to a lesser degree, in cDCs and TLR8 is mainly expressed in macrophages and cDCs [193]. Activation of the TLRs results in the induction of three signaling pathways: the mitogen-activated protein kinases (MAPKs), the nuclear factor kappa-light-chain enhancer of activated B cells (NF-κB) and several interferon regulatory factors (IRFs). The first two pathways result in the expression of cytokines, such as, but not limited to, IL-1β, IL-6, IL-18 and TNF-α, and genes that are related to inflammation and activation of the adaptive immune system. Activation of the IRFs induces the expression of type I interferons, including IFN-α and IFN-β, which are in turn responsible for the expression of interferon-stimulated genes [194]. The RLRs are in the cytoplasm of most cell types, and interaction with viral RNA leads to the activation of the NF-κB and IRF3 pathways. In this way, the RLRs are also responsible for the expression of inflammatory genes, the activation of the adaptive immune system and the production of type I interferons [195].

During viral infection, infected cells produce type I interferons, IFN-α and IFN-β, while infected epithelial cells mainly produce type III interferons. The receptors for these type I interferons are present in most of the porcine cells, implying that the production of IFN-α and IFN-β has a direct and indirect effect on a whole range of cell types [196]. The type I interferons induce an antiviral state by inducing the expression of interferon-stimulated genes in both infected and neighboring cells. Furthermore, innate immune cells increase their antigen presentation and their production of cytokines and chemokines in response to the type I interferons. Most of the cell types can produce IFN-β, while the pDCs are the main producers of IFN-α [197,198].

### 4.3. PRRSV Modulation of the Type I Interferon Response

PRRSV can modulate the innate immune response by inhibiting the type I interferon response, resulting in a downregulation of inflammatory genes and a suppressed activation of the adaptive immune response [192]. The first evidence of the PRRSV modulation resides in the moderate to negligible IFN-α response that is observed during PRRSV infection. It has been shown that the levels of IFN-α in the lung of PRRSV-infected pigs are significantly lower compared to the levels of IFN-α in the lungs of pigs infected with other respiratory viruses, such as PRCV [199]. Moreover, a significant suppression of IFN-α production was observed in pigs co-infected with PRRSV and PRCV compared to pigs infected with PRCV alone [200]. Finally, an in vitro inhibition of IFN-α production was observed in pDCs cultured with PRRSV compared to pDCs cultured with transmissible gastroenteritis virus [185]. However, Baumann et al. (2013) reported that the in vitro inhibition of the pDC function by PRRSV is likely strain-dependent [184].

Several molecular mechanisms behind the PRRSV modulation of the type I interferon responses have been described.

- Several PRRSV proteins can inhibit IRF3, including NSP1β [201], NSP2 [202], NSP11 [203] and the N protein [204]. The main mechanism of inhibition by these proteins includes the blockage of IRF3 phosphorylation and, consequently, a blocked translocation of IRF3 to the nucleus [192].
- Liu et al. (2019) described the downregulation of IRF7 by the PRRSV NSP7 in infected PAMs [205].
- An inhibition of the type I signaling via the RIG-I and MDA5 PRRs has been described for both PRRSV NSP4 and NSP11 [206,207].
- A further modulation of the type I interferon response is achieved by the PRRSV NSP1α. This non-structural protein contributes to the degradation of the cyclic AMP responsive element-binding protein (CBP). Since CBP is an important coactivator for many transcription factors, including the IRFs, its degradation indirectly inhibits the type I interferon response [208,209].
- Liu et al. (2020) described the immunosuppressive function of Sn, which was shown to suppress type I interferon responses by targeting the NF-κB pathway [210]. Further inhibition of the NF-κB pathway has been described for NSP4, which cleaves an essential mediator of the NF-κB pathway, and for NSP1α, NSP2 and NSP11, which interfere with the translocation of NF-κB to the nucleus [211,212,213,214].
- Finally, PRRSV can further modulate the innate immune response by influencing the expression of microRNA (miRNA) [215,216,217,218]. The miRNAs are small non-coding RNAs, typically 19 to 25 nucleotides in length, that play a role during the post-transcriptional gene expression. They can bind to certain target mRNAs, which results in gene silencing [219]. Hicks et al. (2013) performed deep sequencing of in vitro PRRSV-2-infected PAMs at 48 h post-infection and compared the miRNAome of these infected PAMs with the miRNAome of mock-infected PAMs [216]. A significantly different expression of 40 cellular miRNAs was observed in the PRRSV-2 infected PAMs. Furthermore, analysis of the target genes of these differentially expressed miRNAs revealed that most of them targeted genes involved in immune regulation, including the production of cytokines. The upregulation of miR-24-3p [220], miR-30c [221], miR-373 [222] and miR-382-5p [223] has been described during PRRSV infection. These miRNA silence genes are responsible for the type I interferon response. Therefore, the upregulation of these miRNAs by PRRSV inhibits type I interferon production and further facilitates immune escape and replication. Additionally, Zhang et al. (2021) reported the downregulation of miR-218 by PRRSV [224]. This miRNA silences an inhibitor of the type I interferon response, and thus, downregulation of miR-218 results in further inhibition of the interferon response.

In summary, PRRSV can strongly modulate the innate anti-viral immune response through a wide range of mechanisms. Firstly, PRRSV infects and hampers the function of the main phagocytic cell of the innate immune system—the macrophages. Secondly, PRRSV can suppress the type I interferon response, leading to reduced production of IFN-α and IFN-β, further inhibiting the innate immune response. Additionally, the activation of the adaptive immune response is influenced, since naïve T cells require adequate IFN-α to differentiate into IFN-γ-secreting cells (see Section 5.1). Finally, a clear suppression of the cytotoxic activity of NK cells has been described, resulting in the suppressed clearance of infected cells and enhancing infection.

## 5. Modulation of the Adaptive Immune Response

As stated in Section 4, the innate immune response consists of the fast and non-specific first line of defense against invading pathogens. In contrast, the adaptive immune response is slower (days to weeks after entry of the pathogen), highly specific and is induced by the help of the innate immune responses. The adaptive immune responses include pathogen-specific immunologic pathways capable of eliminating both the pathogen and cells infected by the pathogen. Importantly, the adaptive immune system can develop a memory against the specific pathogen, allowing rapid elimination in case of a subsequent infection with the same pathogen. The latter forms the basis of immunization against infectious diseases, which vaccination aims to achieve. The adaptive immune system can be divided into two large components: cell-mediated immunity (CMI), with a critical role for the T-lymphocytes (T cells), and humoral immunity, characterized by antibody production by mature B-lymphocytes (B cells) [225].

### 5.1. T Cells and Cell-Mediated Immunity

**Figure 3:**
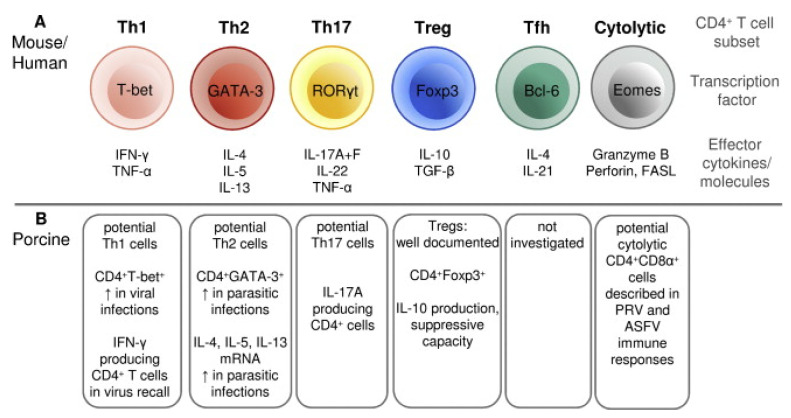
Differentiation of CD4+ T cells in distinct functional subsets of mouse/human compared to pigs (from [230]). (**A**) Differentiation of murine/humane CD4+ T cells in six subsets (adapted from [235]). (**B**) Differentiation of porcine CD4+ T cells in six subsets. T: T-lymphocyte cell; Th: CD4+ T helper cell; Treg: regulatory T cell; Tfh: follicular helper T cell; CD: cluster of differentiation; T-bet: T-box transcription factor 21 expressed in T cell; GATA-3: GATA-3 transcription factor expressed in T cell; RORγt t: retinoic acid-related orphan receptor-γt expressed in T cell; Foxp3: Forkhead box P3 transcription factor expressed in T cell; Bcl-6: transcription factor B cell lymphoma 6 expressed in T cell; Eomes: transcription factor eomesodermin expressed in T cell; IFN: interferon; TNF: tumor necrosis factor; IL: interleukin; TGF: transforming growth factor; FAS: tumor necrosis factor receptor superfamily member 6; FASL: FAS ligand.

T cells are critical components of the immune response against PRRSV and are involved in the maturation and activation of B cells, the induction of a favorable cytokine environment for antigen presentation, the cytotoxic killing of infected cells and the overall regulation of the inflammatory response [226]. Porcine T cells can be divided into two main classes based on the polypeptides that form their T-cell receptor (TCR): the αβ T cells, containing a TCR consisting of α and β chains, and the γδ T cells, containing a TCR consisting of γ and δ chains [227]. In the first class, an additional differentiation in two main subsets is made based on the expression of co-receptors: the CD4+ T cells and CD8+ T cells. The CD4+ T cells can interact with MHC class II molecules and are programmed to elicit helper functions (Th). The CD8+ T cells can interact with MHC class I molecules and are programmed to elicit cytotoxic functions (Tc) [228]. However, a peculiarity for the porcine αβ T cells is the presence of a subpopulation of CD4+ T cells that co-express the CD8α chain: the CD4+CD8α+ T cells [229]. These double-positive T cells are memory T cells, given their capability to proliferate and produce IFN-γ in response to stimulation with a recall antigen, their expression of memory T cell markers and their rapid localization to sites of inflammation [229,230,231]. Like murine and humane CD4+ T cells, a functional differentiation in distinct subsets can be made for the porcine CD4+ T cells based on the expression of specific transcription factors or the production of specific cytokines (Figure 3, adapted from [230]). The CD4+ Th1 cells play a major role in the proinflammatory response to viral infection, producing high levels of IFN-γ. In contrast, the regulatory T cells (Tregs) play a major role in controlling the inflammatory response and are characterized by the production of the immunosuppressive cytokine IL-10. In swine, two subpopulations of Tregs have been described: CD4+CD8-CD25+Foxp3+ and CD4+CD8+CD25+Foxp3+ [232]. Next to the CD4+ subsets visualized in Figure 3, an additional subset has been described by Mair et al. (2016): the NK T cells, a T-cell subset containing characteristics specific for both NK cells (Nkp46+) and T cells (CD3+) [233]. These NK T cells can produce proinflammatory cytokines, such as IFN-γ, and elicit cytolytic activity [234].

The main function of the γδ T cells during infection is the protection against the invading pathogen (cytotoxicity + production of proinflammatory cytokines) and the modulation of both innate and adaptive immune responses through the expression of proinflammatory and regulatory cytokines and chemokines [236]. They cannot be classified as pure adaptive immune cells, given the fact that they can be activated by both their TCR and receptors associated with NK and myeloid cells [191]. They are less specific than the αβ T cells, recognizing a broader range of distinct antigens, and due to their diversified location and rapid response, they are part of the first line of defense [237]. Due to these characteristics, they are usually categorized as part of both innate and adaptive immunity. In pigs, a high frequency of γδ T cells has been described, ranging from 18–47% in blood (depending on age and breed). In sharp contrast, the γδ T cells represent only a small proportion (1–5%) in humans [191]. This suggests that the γδ T cells play a major role in the porcine immune system.

#### Role of T Cells during PRRSV Infection

T cells play a major role in the effector responses to viral infections, including the overall immune activation, the modulation of adequate T and B cell responses, the regulation of intensity and duration of immune response and the development of immune memory. Given this crucial role, it can be hypothesized that the inability to achieve rapid sterilizing immunity and consecutive clearance of PRRSV might be due to inadequate T-cell responses [226]. Despite many efforts of several research groups to investigate T-cell responses during PRRSV infection, the exact role of the T cells remains unclear and warrants further investigation [238]. Nevertheless, when combining all studies, it becomes clear that PRRSV can modulate the T-cell response, which further facilitates infection.

Xiao et al. (2004) reported a decrease in γδ T cell levels throughout infection and the transient induction of PRRSV-specific IFN-γ producing T cells from 14 dpi onwards [239]. A high variability was observed in the T-cell induction, both in time and between infected animals. Additionally, no correlation was found between the levels of PRRSV-specific T cells and the viral load. In contrast, in a study by Kick et al. (2019), a correlation was found between the reduction in viral load from 21 dpi to 35 dpi and the in vitro proliferation rate of isolated Th cells after recall stimulation [240]. However, the authors reported no correlation between the in vivo levels of cytokine-producing Th cells and the viral load during infection. In the same study, a high induction of γδ T cells was observed in the first days post-infection, and γδ T cells, isolated at 28 dpi, were able to proliferate and produce IFN-γ after both homologous and heterologous recall stimulation. This corroborates the study by Olin et al. (2005) in which γδ T cells isolated from PRRSV-infected gilts had higher proliferation rates and IFN-γ production in comparison to γδ T cells isolated from non-infected gilts [241]. Finally, Kick et al. (2019) also reported a high cytotoxic CD8+ response at sites of infection, both in the lungs and in bronchoalveolar fluid [240]. The role of the CD8+ T cells during PRRSV infection warrants further investigation. An early study by Lohse et al. (2004) suggested only a minor role for the CD8+ T cells, since no enhancement of PRRSV infection was observed after the temporary depletion of CD8+ T cells [242]. However, this effect was only investigated until 8 dpi, so no clear conclusions can be drawn from this study. Costers et al. (2009) isolated PBMCs from infected pigs and reported the induced proliferation of CD8+ T cells after in vitro recall from 14 dpi onwards [243]. However, in this study, the CD8+ T cells had no cytotoxic effect on infected alveolar macrophages. Interestingly, Zhang et al. (2016b) reported the suppression of Th17 cells, involved in the response against bacterial infection, by HP-PRRSV as well as the severe depletion of CD4+CD8+ T cells in the early stages of infection [244]. This could potentially be another mechanism in which PRRSV increases the susceptibility to secondary infections.

An additional mechanism of PRRSV modulation is the induction of an immunosuppressive environment by the upregulation of immunosuppressive cytokines, such as IL-10 and TGF-β, and the increased expression of Tregs. The studies conducted by Silva-Campa et al. (2009, 2010, 2012) resulted in valuable insights concerning this topic [245,246,247]. In the first study, the in vitro induction of Tregs by PRRSV-2-infected moDCs was shown, which was mainly dependent on the expression of TGF-β and not on the expression of IL-10 [247]. In the second study, the experiment was repeated, and this time, DCs were infected with four different PRRSV-1 strains. A high production of IL-10 was observed in the DCs, but they were not able to induce Tregs in the co-cultured lymphocytes [246]. In a final in vivo study, they reported increased levels of CD4+CD8+ Tregs from 14 dpi onwards in PRRSV-2-infected pigs, while the total levels of CD4+CD8+ remained constant. Furthermore, a positive correlation was found between the viral load and the induced Tregs in peripheral blood. In a recall assay, the isolated Tregs expressed high levels of TGF-β [245]. Other studies were consistent with these results, reporting the induction of Tregs in blood, lung, and tracheobronchial lymph nodes of PRRSV-infected pigs [248,249]. Finally, several studies reported the induction of apoptosis in the thymus and lymphoid organs, resulting in impaired development of T cells and a reduced immune response as consequence [250,251,252].

### 5.2. B Cells and Humoral Response

B cells are highly specialized cells, exhibiting different functions during the adaptive immune response. They can act as APC and produce cytokines, but most importantly, they are responsible for producing antibodies, which define the humoral immune response. B cell activation requires either a combination of antigen recognition or interaction with CD4+ Th cells (T-cell-dependent activation). Alternatively, B cells can be activated in a T-cell-independent manner involving the cross-linkage of the B cell receptor with polysaccharides or via B cell receptor and TLR co-stimulation. During viral infections, antigen recognition is mediated by the innate APCs, in particular the macrophages and DCs. Following interaction between the B cell and the innate APC, the B cell receptor can internalize the presented antigen. This internalized antigen is subsequently degraded via endocytosis and short peptides of the antigen are presented on the surface of the B cell in the context of MHC class II molecules. CD4+ Th cells that recognize the presented antigen will interact with the B-cell, resulting in a cascade of activation. Finally, the activated B cell can differentiate into a short-lived plasma cell, which results in the first production of antibodies and the initiation of the early adaptive immune response. Alternatively, the activated B cell can proliferate and mature further. These mature B cells can either produce long-living antibodies with a higher affinity or differentiate further into memory B cells. The latter can persist independently of the antigen, meaning that the memory B cell remains viable long after the infection has been cleared. In the case of a second infection, with the same pathogen, the memory B cell will rapidly present antigen to the T cells. This will result in the formation of the memory plasma cell, inducing the rapid production of high-affinity antibodies and a well-coordinated and fast T-cell response to the pathogen [253,254,255].

The produced antibodies, also referred to as immunoglobulins (Igs), consist of two identical heavy and two identical light polypeptide chains. Each polypeptide consists of multiple protein domains, which are further characterized as variable and constant regions. The constant region of the heavy chain determines the class (isotype) of the produced antibody. In pigs, five main Ig isotypes are described, IgM, IgA, IgG, IgE and IgD, each with their specific roles during the immune response. The antibody-mediated immune response against viral infections is mainly orchestrated by IgM, IgA and IgG [253,255,256].

- IgM is associated with the primary immune response. The monomeric IgMs have a lower affinity compared to the other Ig isotypes. However, they will typically form a pentameric complex, consisting of five IgM molecules linked via disulfide bonds. Due to this, the pentameric IgM complex can have multiple interactions with a certain antigen, resulting in a high avidity. The main function of IgM is the coating of the antigen (opsonization), which targets the antigen for destruction [256].
- IgAs are mainly found at mucosal surfaces and secretions, where they are mainly present in the forms of dimers, termed secretory IgA (sIgA). Additionally, they can be present in serum, although at lower concentrations and in the form of monomers. The main function of IgA is the protection of the mucosal surfaces, both by the direct neutralization of pathogens and by the prevention of binding of pathogens to the mucosal surfaces [256].
- IgGs are the dominant isotype, they have the longest serum half-life and play a critical role in the immune response [256]. They can eliminate pathogens, either directly, by neutralization, or indirectly, by activation of the complement cascade [256,257,258]. In pigs, six IgG subclasses were originally reported, based on the cDNA sequencing of the immunoglobulin heavy chain constant region (IGHG) gene [253]. However genomic analyses revealed that the porcine IGHG genes can be classified into nine subclasses, which are variably present in different pig breeds [259]. Therefore, depending on the breed of the pig and the genetic analysis that is used to determine the IGHG genes, a different amount of IgG subclasses can be defined [259].
- IgE plays a minor role in viral infections but is known as a highly potent Ig, typically associated with hypersensitivity and allergy reactions [256].
- Finally, the role of IgD is still under investigation [260]. It was first considered as a vestigial isotype in most mammals, without a role in the antibody-mediated immune response but with a function during the development and activation of B cells [253]. More recent findings indicate that, at least in humans and mice, sIgD could recognize antigens from aerodigestive commensal microbiota, food and pathogens and play a role in the maintenance of mucosal homeostasis in cooperation with IgA and IgG [261,262].

Antibodies can induce the neutralization and/or elimination of the infecting pathogen by several mechanisms. Neutralization occurs when the antibody prevents the virus from functioning, either by blocking the interaction of a certain viral surface protein with the host receptor or by blocking viral components that are critical for the assembly of new viral particles. Additionally, opsonization can occur, in which antibodies bind to the invading pathogen. This can result in the formation of an immune complex. This immune complex can be detected by phagocytic cells destroying the pathogen. Finally, the clearance of pathogens can occur via antibody-dependent cell-mediated cytotoxicity. In this process, the immune complex (antibody bound to pathogen) can be recognized by NK cells, resulting in the production of IFN-γ and the induction of apoptosis [255,263].

#### Role of B Cells and the Humoral Response during PRRSV Infection

The kinetics of antibody production during PRRSV infection have been thoroughly investigated. A robust antibody response is observed from 7 dpi onwards. PRRSV-specific IgMs can be detected at 7 dpi; they reach their peak titers at 14–21 dpi and rapidly decrease to undetectable levels by 40 dpi. PRRSV-specific IgGs can be detected between 7 and 9 dpi; they peak at 21–28 dpi and can persist for several months [264,265,266,267,268,269]. Antibodies targeting the N protein appear first, followed by antibodies against the M protein and finally antibodies against the GP5 protein [267,270]. Additionally, the PRRSV NSP2 contains a large cluster of non-neutralizing B-epitopes, suggesting that this non-structural protein is one of the most immunodominant proteins of PRRSV [271].

The role of the early antibodies is controversial, as they are non-neutralizing and are not correlated to protection [264]. Furthermore, they might even enhance the disease progression by the mechanism of antibody-dependent enhancement (ADE) of infection. ADE is the phenomenon in which binding of non-neutralizing antibodies to viral surface proteins can result in a more efficient entry of the virus into the host cells, increasing the viral infectivity [272]. ADE is a characteristic that has been described for several macrophage-tropic viruses, including feline infectious peritonitis virus [273], Dengue virus [274] and HIV [275]. This has led to the hypothesis that ADE might also be involved in the pathogenesis of PRRSV, especially given the abundance of non-neutralizing antibodies produced during the early stages of PRRSV infection. Several research groups have attempted to investigate this, but the role of ADE in PRRSV pathogenesis remains questionable. The occurrence of ADE has been shown in vitro using PRRSV-infected PAMs [276,277,278,279,280]. In such conditions PRRSV-ADE infection downregulates the levels of type II and III IFNs and facilitates viral replication in PAMs [280]. However, Delputte et al. (2004) did not observe any ADE effect on PRRSV-infected PAMs despite using different dilutions of sera, different purified antibodies and three different PRRSV strains [281]. Additionally, no clear role for ADE in vivo has been found. Yoon et al. (1996) observed a slight increase in viremia in pigs that were administered non-neutralizing antibodies prior to PRRSV challenge compared to pigs that did not receive antibodies [282]. However, no differences were observed in clinical signs or other disease parameters. In a study by Sautter et al. (2019), no evidence was found for ADE using in vivo challenged pigs [283]. In conclusion, although it is possible to observe ADE using in vitro models, this phenomenon likely does not play a role in the pathogenesis of PRRSV.

The appearance of PRRSV-specific neutralizing antibodies (NAbs) is slow (from 28 dpi onwards) and irregular [264,284]. Nevertheless, the protective effect of NAbs against PRRSV infection was demonstrated in passive transfer experiments conducted by Osorio et al. (2002), where naïve pregnant sows receiving PRRSV-specific NAbs (from one animal challenged with a highly abortive strain) were fully protected from reproductive failure in a homologous challenge [285]. Moreover, the absence of infectious viruses detected in the tissues of the sows and the absence of infection in their offspring suggests a sterilizing immunity conferred by the PRRSV-specific NAbs [285]. Additionally, passive transfer experiments conducted by Lopez et al. (2007) described the protection of NAbs in challenged piglets in a dose-dependent manner [286]. However, despite the clear capability of NAbs to prophylactically protect against PRRSV challenge, the exact role of NAbs during PRRSV infection is less understood [182].

- First, the NAbs are not produced rapidly enough to avoid the persistence of viral infection [270,287,288].
- Second, the induction of antibodies directed against the major neutralizing epitope of PRRSV, the GP5 protein, is not sufficient to clear the virus [287].
- Third, PRRSV can persist in infected tissues even after the production of NAbs [97,287,289,290].
- Fourth, most NAbs generated upon infection are strain-specific and do not recognize heterologous viruses [291,292,293].

It has been shown that the neutralizing epitopes of PRRSV, mainly on GP5 and, to a lesser degree, on GP2, GP3 and GP4, are shielded by sugar chains, which are formed via N-linked glycosylation [294,295,296,297,298]. This mechanism of glycan shielding can partially be responsible for the delayed and irregular production of NAbs during PRRSV infection [294,299,300]. The role of glycan shielding in the NAb response against PRRSV has been investigated by several research groups [294,296,297,298]. Vu et al. (2011) described a PRRSV field isolate that was able to induce an atypically fast and robust NAb response [298]. Further analyses showed that this field isolate lacked two N-glycosylation sites, one on the GP3 and one on the GP5 protein, which impaired the glycan shielding of the neutralizing epitopes and could explain the atypical robust NAb response. Wei et al. (2012) conducted (de)glycosylation experiments that showed that glycosylation downstream of the GP5 neutralizing epitopes results in PRRSV resistance to neutralization [296].

Additionally, PRRSV modulation of both the innate immune response (see Section 4) and the T-cell response (see Section 5.1) harms the maturation and activation of the B cells. This, in turn, can potentially impair the production of the high-affinity NAbs.

Despite their controversial efficacy, the investigation on Nabs is supported by their potential interest in vaccination. So far, cross-reactive NAbs, which can recognize and neutralize heterologous viruses including PRRSV-1 and PRRSV-2 isolates, were mostly considered as the result of multiple exposures to genetically diverse PRRSV strains [301]. Animals with broadly cross-reactive neutralizing antibodies against PRRSV have been described in the field with a prevalence estimated at 7% (against PRRSV-1 and PRRSV-2) to 15% (against PRRSV-1 only) in the sow population under field conditions [302]. However, Trible et al. (2015) predicted the existence of a new class of heterologous PRRSV antibody, referred to as a broadly neutralizing antibody (BNAb) [293].

In another research group, the virus sensitivity to neutralization and the cross-reactivity of NAbs was measured in vitro in seroneutralization assays combining 30 individual PRRSV-1 hyperimmune sera and a panel of 39 virus isolates [292]. Both sensitive- and resistant-to-neutralization virus strains were identified, as well as strains inducing NAbs with homologous to heterologous recognition. A second study confirmed the protective effect of the induced broadly reactive NAbs in a challenge experiment with heterologous strains [303].

In a study by Martínez-Lobo et al. (2011), no correlation could be found between the sensitivity to neutralization phenotype of the isolates and the antigenic variability in the known neutralizing epitopes of GP3, GP4 and GP5 or to the number and position of N-linked glycosylation sites that might shield neutralizing epitopes [303]. Kim and Yoon investigated the role of envelope-associated proteins in the cross-neutralization of genetically distinct PRRSV isolates and concluded that ORFs 3 (GP3), 5 (GP5) and 6 (M) were additively responsible for cross neutralization [304]. Another study identified the role of a tyrosine codon deletion in the ORF 6 (Y10) in the cross-reactivity of Nabs, which takes part in a much larger conformational epitope formed by the interaction of GP5, M and other viral structural proteins [293].

## 6. Research Gaps

More than three decades after the first clinical reports of PRRSV, the virus remains abundantly present in the swine production system worldwide, causing devastating production and economic losses. The combination of a high genetic drift and the broad capability to modulate and evade the porcine immune system makes the control of PRRSV a major, and often frustrating, challenge. Despite many efforts to investigate the biology, pathogenesis and immunological properties of the virus, there are still some research gaps that remain unanswered [42].

In terms of PRRSV biology, there is still a lot to be learned from the in-depth function and structure of the viral proteins, as well as from the role of extracellular vesicles (EVs) in the interaction between PRRSV and the pig. The latter are cell-derived vesicles that can contain bioactive molecules that can be delivered to other cells [305]. In the context of PRRSV, it has been shown that EVs are present in the serum of previously infected animals, and these EVs carry PRRSV-specific proteins [306]. However, their exact role in PRRSV pathogenesis remains unclear for now. The recent advancement in whole genome sequencing allows us to better characterize the genetic evolution of the virus and can hopefully aid in predicting how the virus will evolve in the future in terms of genome and pathogenicity.

The immune responses and immune modulation of PRRSV have been extensively studied in the past three decades and will know a new age of discovery with technologies like transcriptomics [307,308,309,310,311,312,313,314]. There is a need for a better understanding of the role of host genetics in innate immune responses. The effect of structural and non-structural proteins of PRRSV is not yet fully understood, neither are the interactions with the antigen-presenting cells. There is still limited knowledge of other cell populations interacting with PRRSV.

The cellular protective mechanisms are not yet fully elicited and there is still no key for predicting protection. The role of neutralizing and non-neutralizing antibodies remains controversial, and more work is necessary to decipher the complexity of broad cross-reactive neutralizing antibody induction and associated epitope recognition.

Research is warranted to fully capture the complex interaction between PRRSV and the pigs’ immune system. The latter has a direct consequence for the development of future vaccines.

## 7. Methodology

This review was conducted through a manual search across several academic databases, including PubMed, Google Scholar and Scopus, using keywords such as “Porcine Reproductive and Respiratory Syndrome”, “PRRSV”, “Porcine Immunology”, “innate immunity”, “adaptative immunity” and Boolean operators, with focus on recent publications. Chapters from reference books, review papers and original research articles that specifically addressed the sections of this review were used to construct the comprehensive review. Titles and abstracts were first screened to filter out irrelevant studies. Full-text articles were then reviewed to ensure they met our inclusion criteria. Finally, we also examined the reference lists of selected articles to identify additional relevant studies that might not have been captured in our initial database search and proceeded via the “snowball” method.

## 8. Conclusions

Porcine reproductive and respiratory syndrome (PRRS) remains, since its emergence in the Eighties, a worldwide health problem in many pig herds. Vaccination is widely used to control the severity of clinical signs and the economic impact of the disease. Nevertheless, it is not sufficient as a single measure to achieve virus eradication.

On the contrary, PRRSV has a remarkable capacity to adapt to its porcine host, developing strategies to inhibit, evade and overcome the host’s immunity in all its components, namely innate and adaptive, humoral and cellular. Many of these topics are poorly understood and only partially explored, such as (1) the mechanisms to inhibit the antiviral pathways of the innate immune system, (2) the mechanisms to evade the adaptive immune system, (3) the characterization of cross-reactive neutralizing epitopes and (4) the risks associated with the predominance of non-neutralizing antibodies. Moreover, some of these interactions and mechanisms have been studied based on specific viral strains. Given the huge diversity of PRRSV, results cannot always be extrapolated to all strains, and different studies may lead to contradictory and confusing results.

A better understanding of the interaction mechanisms between PRRSV and the immune system is required, considering the genetic diversity of the virus. This knowledge is essential for developing new generations of vaccines with higher protective efficacy.
